# Urethral Sounds

**Published:** 1869-03-15

**Authors:** 


### *Urethral Sounds.*

Bliss & Sharp, 144 Lake street, furnish a full case (12) with gauge and steel sounds, for $20. Same, silver-plated, $26. See their advertisement this number, for answers to further inquiries from correspondents.

